# FDX1 Regulates the Phosphorylation of ATM, DNA-PKcs Akt, and EGFR and Affects Radioresistance Under Severe Hypoxia in the Glioblastoma Cell Line T98G

**DOI:** 10.3390/ijms26073378

**Published:** 2025-04-04

**Authors:** Takuma Hashimoto, Kazuki Tsubota, Khaled Hatabi, Yoshio Hosoi

**Affiliations:** Laboratory of Radiation Biology, Tohoku University School of Medicine, 2-1 Seiryo-machi, Aoba-ku, Sendai 980-8575, Miyagi, Japan; t.hashimoto@tohoku.ac.jp (T.H.); hatabi.khaled.s4@dc.tohoku.ac.jp (K.H.)

**Keywords:** radiation sensitivity, hypoxia, FDX1, ATM, DNA-PKcs, Akt, EGFR

## Abstract

Hypoxic cells exhibit radioresistance, which is associated with poor prognosis in cancer patients. Understanding the molecular mechanisms underlying radioresistance in hypoxic tumor cells is crucial for improving radiotherapy efficacy. In this study, we examined the role of FDX1 in regulating cellular responses to severe hypoxia in glioblastoma cell lines T98G and A172. We found that FDX1 expression was upregulated under severe hypoxia, and its knockdown reduced the hypoxia-induced activation of key radioresistance factors and cellular survival mechanisms, including ATM, DNA-PKcs, Akt, and EGFR. FDX1 knockdown also sensitized T98G cells to radiation under severe hypoxia. Furthermore, FDX1 was found to regulate HIF-1α protein level, while HIF-1α did not regulate FDX1 expression. These results suggest that FDX1 may be a novel therapeutic target to overcome radioresistance in glioblastoma under severe hypoxia.

## 1. Introduction

Solid tumors often exhibit abnormal vascular structures, leading to insufficient oxygen supply to cells distant from blood vessels [1,2]. These hypoxic regions within the tumor microenvironment are associated with radioresistance, reducing the effectiveness of radiotherapy [3,4,5]. Tumor cells in these regions exhibit reduced sensitivity to radiation, a phenomenon commonly referred to as the “Oxygen Effect” [2,6,7]. Although this phenomenon is well-established, the biological mechanisms underlying radioresistance due to severe hypoxia remain poorly understood.

We have previously shown that severe hypoxia (with oxygen concentrations below 0.1%) increases the expression and phosphorylation of DNA damage response proteins ATM and DNA-PKcs, essential for repairing double-strand breaks in DNA [8,9,10]. Furthermore, we discovered that ATM expression and activation under severe hypoxia are regulated through the energy sensor AMPK and the stress-responsive transcription factor Sp1, contributing to cellular radioresistance in a glioblastoma multiforme (GBM) cell line T98G [9]. However, while suppression of AMPKα significantly reduced radioresistance, it did not fully restore radiosensitivity to levels observed under normoxia. DNA-PKcs and the survival signaling factor Akt, enhanced under severe hypoxia, also remained highly active, independent of the AMPK/Sp1/ATM pathway.

As the energy sensors AMPK and Sp1 do not fully explain the mechanisms underlying hypoxia-induced radioresistance, we sought to identify additional factors involved in the metabolic response to hypoxia and the regulation of DNA-PKcs and Akt. Recent studies have shown that FDX1 is significantly upregulated in GBM and is associated with poor prognosis [11]. FDX1 plays a critical role in protein lipoylation (lipoic acid modification), which is essential for the function of mitochondrial enzyme complexes and vital for energy metabolism [12,13]. Furthermore, it has been reported that while hypoxia can rescue the growth phenotype of cells with either FDX1 or lipoyl synthase knockout, the lipoylation process in these cells is not restored, arguing against an alternative biosynthetic route or salvage pathway for lipoate under hypoxia [14]. These findings led us to investigate the role of FDX1 in regulating cell survival, including its effect on radiosensitivity under severe hypoxia.

In this study, we aimed to determine whether FDX1 contributes to radioresistance in human glioblastoma cells under severe hypoxia and whether it regulates key factors such as ATM, DNA-PKcs, Akt, and EGFR, which are highly expressed in cancer cells. Our results indicate that FDX1 is a crucial regulator of these factors and acts upstream of HIF-1α and AMPKα. These findings suggest that targeting FDX1 may be a promising strategy for overcoming radiation resistance in glioblastoma under severe hypoxia.

## 2. Results

### 2.1. Effects of Severe Hypoxia on FDX1 Expression and the Influence of FDX1 Knockdown on Akt and EGFR

**Figure 1:**
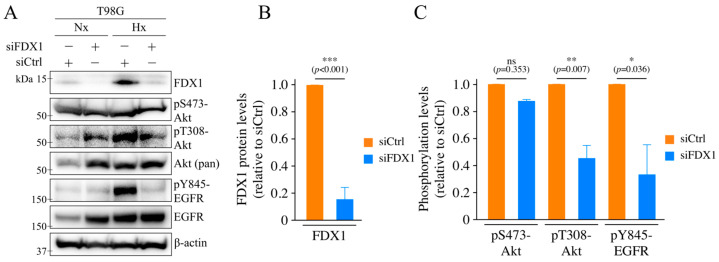
Induction of FDX1 expression under severe hypoxia and the effects of FDX1 knockdown on the activation of Akt and EGFR. (**A**) The effects of severe hypoxia on the expression and/or phosphorylation of FDX1, Akt, and EGFR were investigated in T98G cells with FDX1 knockdown. After 48 h of siRNA treatment (siCtrl or siFDX1), cells were cultured for 18 h under either severe hypoxia or normoxia and then subjected to Western blot analysis using the indicated antibodies. β-actin served as an internal control. (**B**) Quantification of the effect of siFDX1 on FDX1 expression levels under severe hypoxia (with siCtrl set to 1). (**C**) Quantification of the effect of siFDX1 on the phosphorylation levels of Akt and EGFR under severe hypoxia (with siCtrl set to 1). Data are expressed as mean ± SD from three independent experiments (*n* = 3); ns: not significant, * *p* < 0.05, ** *p* < 0.01, *** *p* < 0.001 (Student’s *t*-test). “Nx” represents normoxia, and “Hx” represents severe hypoxia.

We first examined the effects of severe hypoxia on the expression of FDX1 in human glioblastoma cell lines. T98G cells were cultured under hypoxia (<0.1% oxygen) for 18 h. The expression of FDX1 was significantly upregulated compared to normoxia (Figure 1A). FDX1 expression was suppressed using siRNA targeting FDX1 (siFDX1), and the hypoxia-induced increase in FDX1 expression was significantly decreased (Figure 1A,B and Figure S1). To verify whether this response is specific to T98G cells, we treated A172 cells with hypoxia and analyzed FDX1 expression. As in T98G cells, FDX1 expression was upregulated under severe hypoxia in A172 cells, and this increase was suppressed by FDX1 knockdown (Figure S2).

Next, we investigated whether FDX1 expression under severe hypoxia plays a crucial role in cell survival by analyzing the effects of FDX1 knockdown on the expression and activity of epidermal growth factor receptor (EGFR) and Akt. EGFR and Akt are known contributors to radioresistance and cell survival [15,16,17]. Phosphorylation of EGFR at tyrosine 845 (Y845), an activation site for EGFR, was significantly enhanced under hypoxia (Figure 1A). FDX1 knockdown reduced both the expression of EGFR and its phosphorylation under hypoxia (Figure 1A,C). Similarly, phosphorylation of Akt at threonine 308 (T308) and serine 473 (S473), key activation sites, was increased under hypoxia (Figure 1A). However, FDX1 knockdown suppressed phosphorylation at T308, while phosphorylation at S473 remained unaffected (Figure 1A,C).

### 2.2. Effects of FDX1 Knockdown on Radioresistance Under Severe Hypoxia

**Figure 2:**
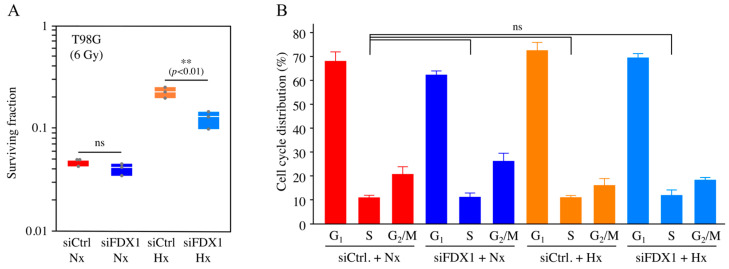
Effects of FDX1 knockdown on radioresistance and cell cycle distribution under severe hypoxia. Radiosensitivity under severe hypoxia was assessed in T98G cells with FDX1 knockdown. After 48 h of siRNA treatment (siCtrl or siFDX1), cells were cultured for 18 h under either severe hypoxia or normoxia. Subsequently, cells were irradiated with 0 or 6 Gy X-rays while maintaining hypoxia. After irradiation, cells were cultured for an additional 4 h under the same conditions before being used for the colony formation assay. (**A**) Box plots showing the colony formation assay results (6 Gy). Values are presented as mean ± SD (*n* = 3). ns: not significant, ** *p* < 0.01 (Student’s *t*-test). Results are also shown in Figure S3. (**B**) The effect of FDX1 knockdown on cell cycle distribution in T98G cells under severe hypoxia was analyzed by flow cytometry. Values are presented as mean ± SD from three independent experiments (*n* = 3); ns: not significant (Dunnett’s test). “Nx” represents normoxia, and “Hx” represents severe hypoxia.

As FDX1 knockdown under severe hypoxia decreased EGFR and Akt phosphorylation (Figure 1), we then explored whether FDX1 contributes to radioresistance. After FDX1 knockdown, cells were irradiated with X-rays while maintaining hypoxia, and cell survival was assessed using the colony formation assay. As shown in Figure 2A and Figure S3, colony numbers in irradiated T98G cells were significantly decreased in the siFDX1 group compared to the control group (siCtrl) under severe hypoxia. These findings suggest that FDX1 is crucial in radioresistance in human glioblastoma cells.

### 2.3. Effects of FDX1 Knockdown on Cell Cycle Distribution Under Severe Hypoxia

Generally, radiation sensitivity is low during the S phase. To determine whether the radiosensitization induced by FDX1 knockdown under severe hypoxia is due to changes in cell cycle distribution, we examined the effect of FDX1 knockdown on the cell cycle. FDX1 knockdown slightly increased the S phase fraction—from 11.2% (siCtrl + Nx) to 11.4% (siFDX1 + Nx) and from 11.3% (siCtrl + Hx) to 12.2% (siFDX1 + Hx) (Figure 2B). There was no statistically significant difference in the S phase fraction when comparing the siCtrl + Nx group with the siFDX1 + Nx, siCtrl + Hx, and siFDX1 + Hx groups. These results suggest that the radiosensitization caused by FDX1 knockdown under severe hypoxia is not attributable to changes in cell cycle distribution.

Furthermore, we evaluated the effect of FDX1 knockdown on cell proliferation. Under normoxia, the siFDX1 group showed a significant decrease in cell number (Figure S4), suggesting that FDX1 knockdown affects cell proliferation and viability under normoxic conditions. In contrast, under severe hypoxia, no statistically significant difference in cell number was observed between the siCtrl and siFDX1 groups (Figure S5), indicating that the effect of FDX1 knockdown on cell proliferation and viability is limited under severe hypoxia.

### 2.4. Effects of FDX1 Knockdown on DNA Double-Strand Break Repair Enzymes Under Severe Hypoxia

**Figure 3:**
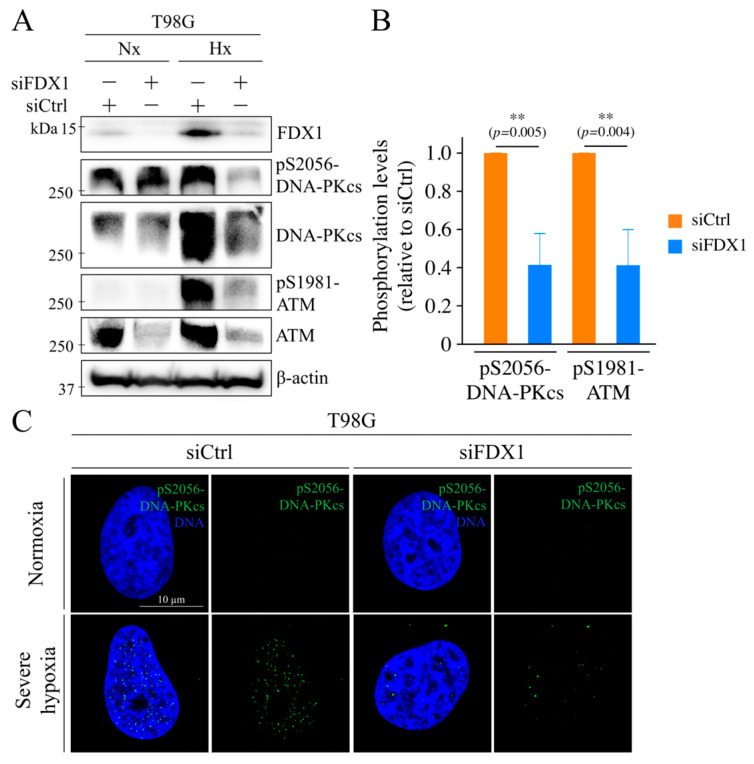
Effects of FDX1 knockdown on the expression and activation of DNA double-strand break repair enzymes under severe hypoxia. The effects of severe hypoxia on the expression and phosphorylation of DNA-PKcs and ATM were examined in T98G cells with FDX1 knockdown. After 48 h of siRNA treatment (siCtrl or siFDX1), cells were cultured for 18 h under either severe hypoxia or normoxia. (**A**) Cells were then processed for Western blot analysis using the indicated antibodies. β-actin was used as an internal control. (**B**) Quantification of the effect of siFDX1 on the phosphorylation levels of DNA-PKcs and ATM under severe hypoxia (with siCtrl set to 1). Data are expressed as mean ± SD from three independent experiments. ** *p* < 0.01 (Student’s *t*-test). “Nx” represents normoxia, and “Hx” represents severe hypoxia. (**C**) Representative confocal microscopy images showing the effect of FDX1 knockdown on the subcellular localization of phosphorylated DNA-PKcs under severe hypoxia.

Next, we examined the effects of FDX1 knockdown on DNA double-strand break (DSB) repair enzymes. First, we analyzed ATM, a key enzyme involved in DSB repair [18]. Phosphorylation at serine 1981 (S1981), an important autophosphorylation site for ATM activation [12], was increased under hypoxia, and this increase was significantly suppressed by FDX1 knockdown (Figure 3A,B). Additionally, ATM expression was upregulated under hypoxia and reduced by FDX1 knockdown. Next, we analyzed DNA-PKcs, another key DSB repair enzyme [19]. Phosphorylation at serine 2056 (S2056), an autophosphorylation site for DNA-PKcs, was enhanced under hypoxia, and this enhancement was significantly suppressed by FDX1 knockdown (Figure 3A,B). We further investigated the impact of FDX1 knockdown on the subcellular localization of phosphorylated DNA-PKcs (S2056) under severe hypoxia. Nuclear localization of S2056-DNA-PKcs, which was detected under hypoxia, was reduced following FDX1 knockdown (Figure 3C). These findings suggest that FDX1 plays a role in regulating the activation and subcellular localization of DSB repair enzymes DNA-PKcs under severe hypoxia, and it also regulates ATM.

### 2.5. Effect of FDX1 Knockdown on ATM, DNA-Pkcs, and Akt Activation in Response to Radiation Exposure Under Severe Hypoxia

We demonstrated that FDX1 knockdown under severe hypoxia significantly suppressed the phosphorylation of ATM, DNA-PKcs, and Akt, as shown in Figure 1 and Figure 3. Additionally, colony formation assays indicated that FDX1 knockdown significantly decreased radioresistance in T98G cells (Figure 2). Considering that ATM, DNA-PKcs, and Akt are key enzymes activated by both severe hypoxia and radiation, we further examined how FDX1 knockdown affects the activation of these enzymes after radiation exposure.

**Figure 4:**
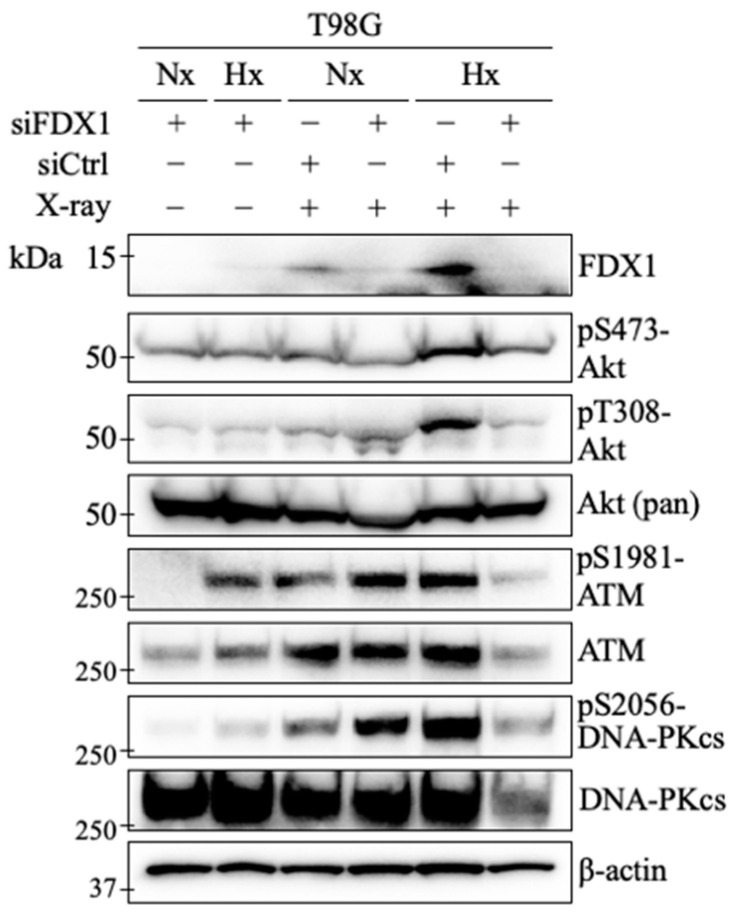
Effects of FDX1 knockdown on ATM, DNA-PKcs, and Akt expression and activation following X-ray irradiation under severe hypoxia. The impact of X-ray irradiation under severe hypoxia on the expression and phosphorylation of Akt, DNA-PKcs, and ATM was examined in T98G cells with FDX1 knockdown. After 48 h of siRNA treatment (siCtrl or siFDX1), the cells were cultured for 18 h under either severe hypoxia or normoxia. Following irradiation with 6 Gy of X-rays, the cells were cultured for 4 h and then subjected to Western blot analysis with antibodies indicated. β-actin as an internal control. “Nx” represents normoxia, while “Hx” represents severe hypoxia.

It is crucial that cells remain in a hypoxic state during radiation exposure to observe enhanced radioresistance due to the oxygen effect [20]. To maintain severe hypoxia during X-ray exposure, T-25 flasks were sealed immediately after hypoxia treatment and irradiated with 6 Gy X-rays. In the radiation exposure groups with siCtrl, FDX1 expression was confirmed in T98G cells (Figure 4). Under severe hypoxia, radiation exposure induced a notable increase in phosphorylation at S1981, which was significantly suppressed by FDX1 knockdown. Similarly, phosphorylation of DNA-PKcs at S2056 was increased under severe hypoxia in irradiated T98G cells. This increase was significantly reduced by FDX1 knockdown. Under severe hypoxia, Akt phosphorylation at T308 and S473 was significantly increased, and FDX1 knockdown effectively inhibited these increases. These findings suggest that FDX1 knockdown suppresses the activation of ATM, DNA-PKcs, and Akt in irradiated T98G cells under severe hypoxia.

### 2.6. Effects of FDX1 Knockdown on HIF-1α Expression Under Severe Hypoxia

**Figure 5:**
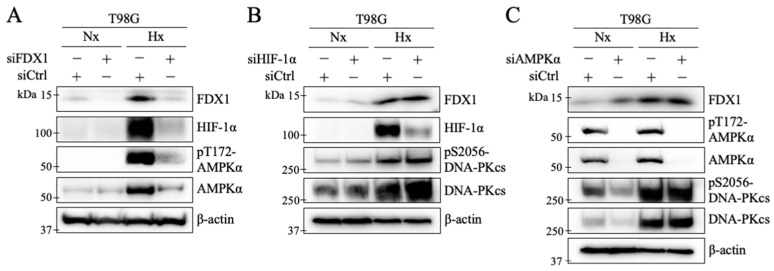
Effects of FDX1, HIF-1α, and AMPKα knockdown on the expression of each under severe hypoxia. (**A**) The effects of severe hypoxia on HIF-1α protein levels and AMPKα expression were examined using T98G cells with FDX1 knockdown. (**B**) The effect of HIF-1α knockdown on FDX1 expression. (**C**) The effect of AMPKα knockdown on FDX1 expression. β-actin was used as an internal control. “Nx” represents normoxia, and “Hx” represents severe hypoxia.

HIF-1α is a transcription factor under hypoxia, triggering various cellular responses [21,22,23]. To investigate the relationship between HIF-1α and FDX1, we analyzed HIF-1α expression following FDX1 knockdown. Under severe hypoxia, HIF-1α protein levels increased, and this hypoxia-induced increase in HIF-1α expression was suppressed by FDX1 knockdown (Figure 5A). Next, we examined the effect of HIF-1α knockdown on FDX1 expression under severe hypoxia. Despite HIF-1α knockdown, the increase in FDX1 expression remained unaffected (Figure 5B). Additionally, no change was observed in the phosphorylation of DNA-PKcs at S2056 under severe hypoxia with siHIF-1α, indicating that the hypoxia-induced increase in phosphorylation was sustained (Figure 5B). These findings suggest that FDX1 acts upstream of HIF-1α under severe hypoxia.

### 2.7. Effects of FDX1 Knockdown on AMPKα Activation Under Severe Hypoxia

Previous studies have shown that ATM activity under hypoxia is regulated by the energy sensor AMPKα [9]. In this study, FDX1 also regulates ATM under hypoxia (Figure 3). To further investigate the relationship between FDX1 and AMPKα under severe hypoxia, we examined their interaction in T98G cells. In cells with FDX1 knockdown, the expression and phosphorylation at threonine 172 (T172) of AMPKα were significantly suppressed by FDX1 knockdown (Figure 5A). FDX1 expression and phosphorylation of DNA-PKcs at S2056 were not reduced by AMPKα knockdown (Figure 5C). These findings suggest that FDX1 acts upstream of AMPKα under severe hypoxia.

## 3. Discussion

**Figure 6:**
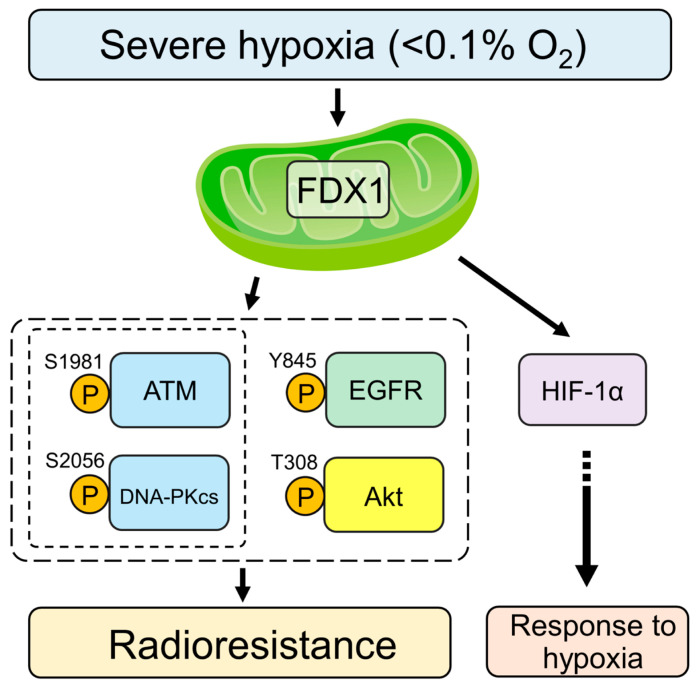
Schematic model of the molecular mechanism of radioresistance mediated by FDX1 under severe hypoxia.

In this study, we demonstrated that in human glioblastoma-derived cell lines, T98G and A172, under severe hypoxia (with oxygen concentrations below 0.1%), FDX1 regulates the activation of key radioresistance factors, including ATM, DNA-PKcs, Akt, and EGFR (Figure 1, Figure 3, and Figure 4). Moreover, FDX1 knockdown significantly sensitized T98G cells to radiation (Figure 2A). It should be noted that the colony formation assay does not necessarily reflect cell proliferative capacity; indeed, when we examined cell proliferation, FDX1 knockdown had no statistically significant effect under severe hypoxia. Previous studies have linked the molecular mechanisms responsible for radioresistance under severe hypoxia to the regulation of ATM via the AMPKα/Sp1 pathway [9]. However, it has also been reported that activation of DNA-PKcs and Akt under severe hypoxia occurs independently of AMPKα, and the regulatory mechanisms for these pathways were previously unclear. In this study, we identified FDX1 as a novel upstream regulator of both DNA-PKcs and Akt under severe hypoxia (Figure 6), providing new insights into their regulation that do not rely on AMPKα. These findings suggest that FDX1 contributes to radioresistance through non-AMPKα-dependent signaling pathways, including DNA-PKcs and Akt, which may represent key mechanisms controlling radioresistance under severe hypoxia.

HIF-1α is recognized as a critical regulator of cellular responses to hypoxia [21,22,23]. Our study demonstrated that FDX1 knockdown led to a reduction in HIF-1α expression levels in T98G cells under severe hypoxia (Figure 5A). In contrast, HIF-1α knockdown did not affect FDX1 expression or the phosphorylation of DNA-PKcs (Figure 5B). To date, only a few hypoxia-induced processes are independent of HIF-1 [24,25]. Our findings suggest that FDX1 acts as an upstream factor, regulating DNA-PKcs and modulating HIF-1α expression levels through distinct pathways in glioblastoma cells under severe hypoxia.

Previous studies have shown that AMPKα knockdown significantly reduces radioresistance in human glioblastoma-derived cells under hypoxia, although the suppression did not reach the levels observed under normoxia [9]. Similarly, this study demonstrated that FDX1 knockdown significantly reduced radioresistance; however, the effect did not reach the levels observed under normoxia. While FDX1 knockdown reduced the expression and phosphorylation of radioresistance-related factors under severe hypoxia, residual levels of FDX1, ATM, DNA-PKcs, and Akt activity were still detected by Western blot (Figure 4). This residual activity may explain why radioresistance was not entirely suppressed. Moreover, radioresistance under severe hypoxia is likely regulated by multiple pathways, suggesting that a more effective strategy for overcoming radioresistance could involve the robust dual inhibition of FDX1 and AMPKα.

Interestingly, FDX1 expression varies across different tumor types, and high expression of FDX1 is predicted to correlate with improved patient prognosis [26,27]. In thyroid cancer, FDX1 knockdown has been shown to suppress cell death induced by cuproptosis [28]. Decreased FDX1 expression has been reported to promote the progression of hepatocellular carcinoma and activate the PI3K/AKT pathway [29]. On the other hand, FDX1 is essential for lipoyl cofactor synthesis in cells under hypoxia and supports cell growth in low-oxygen environments [13]. In this study, we demonstrate that FDX1 knockdown sensitizes T98G cells to radiation under severe hypoxia. Given these findings, the role of FDX1 may represent a specific response influenced by variations in tumor tissue and the microenvironment, including hypoxia, in cancer cells.

Future studies aimed at inhibiting FDX1 could further elucidate its role in regulating radioresistance. In this study, FDX1 expression was inhibited using siRNA; however, it will be essential to investigate the effects of specific inhibitors for potential clinical application. Elesclomol is a mitochondrion-targeting copper ionophore developed as a chemotherapeutic agent [30]. It directly targets FDX1 and inhibits FDX1-mediated Fe-S cluster biosynthesis, thereby inducing copper-dependent cell death in human breast cancer and lung adenocarcinoma cells [31]. While this compound has primarily been studied for its role in copper-induced cell death (cuproptosis), the exact mechanism by which it suppresses cancer remains unclear [32]. Furthermore, the effects of Elesclomol on radioresistance under severe hypoxia have not yet been explored. Investigating FDX1 inhibition using Elesclomol could provide valuable insights into radioresistance under hypoxic conditions and offer an important step toward potential clinical applications.

In conclusion, we demonstrated that FDX1 knockdown suppresses cellular radioresistance under severe hypoxia by regulating ATM, DNA-PKcs, Akt, and EGFR expression in the human glioblastoma cell line T98G. Glioblastoma (GBM) is the most aggressive primary brain tumor in adults, with a poor prognosis [33]. Hypoxic microenvironments in GBM are strongly associated with tumor growth, progression, and resistance to chemotherapy and radiation therapy [34]. In treating GBM, the previously reported AMPK/Sp1/ATM pathway and the FDX1-mediated DNA-PKcs/Akt regulatory mechanism may serve as a promising selective target to overcome radioresistance under severe hypoxia.

## 4. Materials and Methods

### 4.1. Cell Lines and Culture Conditions

Human glioblastoma (GBM) cell lines T98G and A172 were obtained from the American Type Culture Collection (Rockville, MD, USA). The cells were cultured in Dulbecco’s Modified Eagle Medium (DMEM, Low Glucose) (08456-36) (Nacalai Tesque, Kyoto, Japan) supplemented with 10% fetal bovine serum (SH30396.03) (Cytiva, Tokyo, Japan) and 1% Penicillin–Streptomycin Mixed Solution (09367-34) (Nacalai Tesque). Cells were cultured at 37 °C in a 5% CO_2_ incubator.

### 4.2. Hypoxia Treatment

Hypoxia treatment was performed as described previously [5,6]. Cells were incubated in a Modular Incubator Chamber (MIC-101) (Billups-Rothenberg, Del Mar, CA, USA) at 37 °C under a gas mixture of 95% N2 and 5% CO_2_. The chamber was sealed, and oxygen concentration was maintained below 0.1%, as monitored by an oxygen concentration monitor (JKO-O2 Ver. 3, Ichinen Jikco, Nagoya, Japan).

### 4.3. siRNA-Mediated Gene Knockdown

According to the manufacturer’s protocol, gene silencing was achieved using Lipofectamine RNAiMAX Reagent (13778150) (Invitrogen, Carlsbad, CA, USA). A mixture of Lipofectamine RNAiMAX, siRNA, and Opti-MEM (31985-070) (Gibco, Grand Island, NY, USA) was prepared and incubated for 20 min at room temperature. After this, the mixture was added to the cell culture medium, and cells were incubated at 37 °C for 48 h. siRNA targeting FDX1 (siFDX1; 5′-GAUCUGGCAUAUGGACUAA-3′) (SO-3166656G) (Dharmacon, Lafayette, CO, USA) was used, while non-targeting siRNA (D-0018-02) (Dharmacon) was used as a control.

### 4.4. X-Ray Irradiation

After hypoxia treatment, cells in T-25 flasks were irradiated while maintaining hypoxia. X-ray irradiation was conducted using an X-ray generator (M-150WE-H) (SOFTEX, Tokyo, Japan). Cells were exposed to a dose of 6 Gy at 130 kV, 8 mA, with a dose rate of 0.564 Gy/min, using a 0.5 mm thick aluminum filter. After irradiation, cells were returned to a 37 °C incubator for 4 h to assess the impact on DNA damage response.

### 4.5. Colony Formation Assay

Hypoxia treatment was performed as described previously [6]. After X-ray irradiation, cells were detached from T-25 flasks and counted. The cell suspension was mixed with trypan blue for dead cell counting. The cell density was adjusted to 100–12,000 cells/dish, and the cells were plated in 60 mm dishes. After incubation at 37 °C for 14–21 days, colonies were fixed with 10% formaldehyde and stained with 0.01% crystal violet. The plating efficiency (PE) was calculated by dividing the number of colonies containing more than 50 cells by the number of cells plated. The surviving fraction (SF) was calculated by dividing irradiated cells’ PE by non-irradiated control cells’ PE. Three independent experiments were performed.

### 4.6. Western Blotting

The protein content of the samples was quantified using a BCA protein assay kit (Pierce BCA Protein Assay Kit) (Thermo Fisher Scientific K.K., Tokyo, Japan) according to the manufacturer’s instructions. For Western blotting, proteins were mixed with SDS sample buffer containing 12% (*vol*/*vol*) 2-mercaptoethanol and 1% (*vol*/*vol*) loading dye (bromophenol blue and crystal violet). Samples were resolved by SDS-PAGE (5–20% gradient gel, E-D520L, ATTO Technology, Tokyo, Japan) and transferred to a polyvinylidene fluoride (PVDF) membrane (EMD Millipore, Billerica, MA, USA). The membranes were blocked for 60 min using 5% skim milk or PVDF Blocking Reagent (NYPBR01, NOF Corporation, Tokyo, Japan). PVDF Blocking Reagent was used for phospho-specific antibodies. After blocking, membranes were incubated overnight at 4 °C with primary antibodies, followed by secondary antibody incubation at room temperature for 60 min. Antibody diluents were prepared using Can Get Signal Solution 1 and2 (Toyobo, Osaka, Japan). Primary antibodies (Supplementary Table S1) were diluted 1:1000, and secondary antibodies were diluted 1:3000. Detection was performed using chemiluminescent reagents (Chemi-Lumi One Ultra, Nacalai Tesque) and the ChemiDoc XRS Plus system (Bio-Rad, Hercules, CA, USA). Precision Plus Protein Dual Color Standards (#1610374, Bio-Rad) were used for molecular weight estimation. To remove primary and secondary antibodies from the PVDF membrane, Western blot Stripping Buffer (Toyobo) was used. After stripping, membranes were rinsed in wash buffer and re-probed with antibodies. At least two to three independent experiments were performed. The band intensities from the Western blot analyses, obtained from three independent experimental samples, were quantified using ImageJ (version 1.54g). For biological reproducibility, see Supplementary Figure S1.

### 4.7. Proliferation Assay

To evaluate cell proliferation, cells were seeded at a density of 3000 cells per well in a 24-well with siRNA knockdown. For the hypoxia experiment, cells were cultured under normoxia for 12 h and then transferred to hypoxia. The assay was performed for up to 72 h under normoxia experiment and for 48 h under hypoxia experiment. Cells were washed twice with phosphate-buffered saline (PBS (−)) and detached from the culture surface by trypsinization. The total cell number was determined using a particle counter (Coulter Counter Z1, Beckman Coulter, Brea, CA, USA), and dead cells were excluded using the trypan blue dye exclusion method. Two independent experiments were performed.

### 4.8. Cell Cycle Analysis

Cell cycle analysis was performed as previously described [12]. Cells were trypsinized, fixed, and stained with propidium iodide (PI) (Dojindo Molecular Technologies, Inc., Kumamoto, Japan) along with 10 μg/mL of RNase A (Nippon Gene, Tokyo, Japan). The cells were then incubated at room temperature for 30 min. DNA content in each cell was analyzed using a FACSCanto II flow cytometer system (Becton, Dickinson, and Company, Franklin Lakes, NJ, USA). Three independent experiments were performed.

### 4.9. Cellular Localization Analysis

T98G cells were cultured on Microscope Glass Slide (SCS-NO4, Matsunami, Osaka, Japan) and subjected to hypoxia treatment. After hypoxia treatment, the cells were fixed with 4% formaldehyde for 20 min and permeabilized with 0.5% Triton X-100 for 15 min. The cells were then blocked with 10% goat serum for 10 min and incubated with anti-phospho-S2056 DNA-PKcs antibody (Abcam, Cambridge, MA, USA, ab124918, 1/250 dilution) for 1 h, followed by incubation with Alexa Fluor 488 goat anti-rabbit IgG (Molecular Probes, Eugene, OR. A-11008, 1/500 dilution) for 1 h. Nuclear DNA was counterstained with 4′,6-diamidino-2-phenylindole (DAPI, Dojindo, Kumamoto, Japan). All reactions were performed at room temperature (RT). Fluorescent signals were observed using a confocal microscope (LSM800, Carl Zeiss, Oberkochen, Germany). The acquired images were analyzed as AiryScan images using ZEN software (version 2.3 Blue).

### 4.10. Statistical Analysis

For pairwise comparisons, Student’s *t*-test or Dunnett’s test was performed based on the equality of variance between samples, as determined by an F-test. Statistical significance is indicated as * *p* < 0.05, ** *p* < 0.01, and *** *p* < 0.001.

## Data Availability

The authors confirm that the data supporting the findings of this study are available within the article.

## Supplementary Materials

The following supporting information can be downloaded at: https://www.mdpi.com/article/10.3390/ijms26073378/s1.
