# Supplementary material for: FDX1 Regulates the Phosphorylation of ATM, DNA-PKcs Akt, and EGFR and Affects Radioresistance Under Severe Hypoxia in the Glioblastoma Cell Line T98G
Source: Int J Mol Sci. 2025 Apr 4;26(7):3378. doi: 10.3390/ijms26073378 (PMC11990063; doi:10.3390/ijms26073378)
Supplement: Supplementary file 1 [file ijms-26-03378-s001.zip › ijms-3517129-supplementary.pdf]

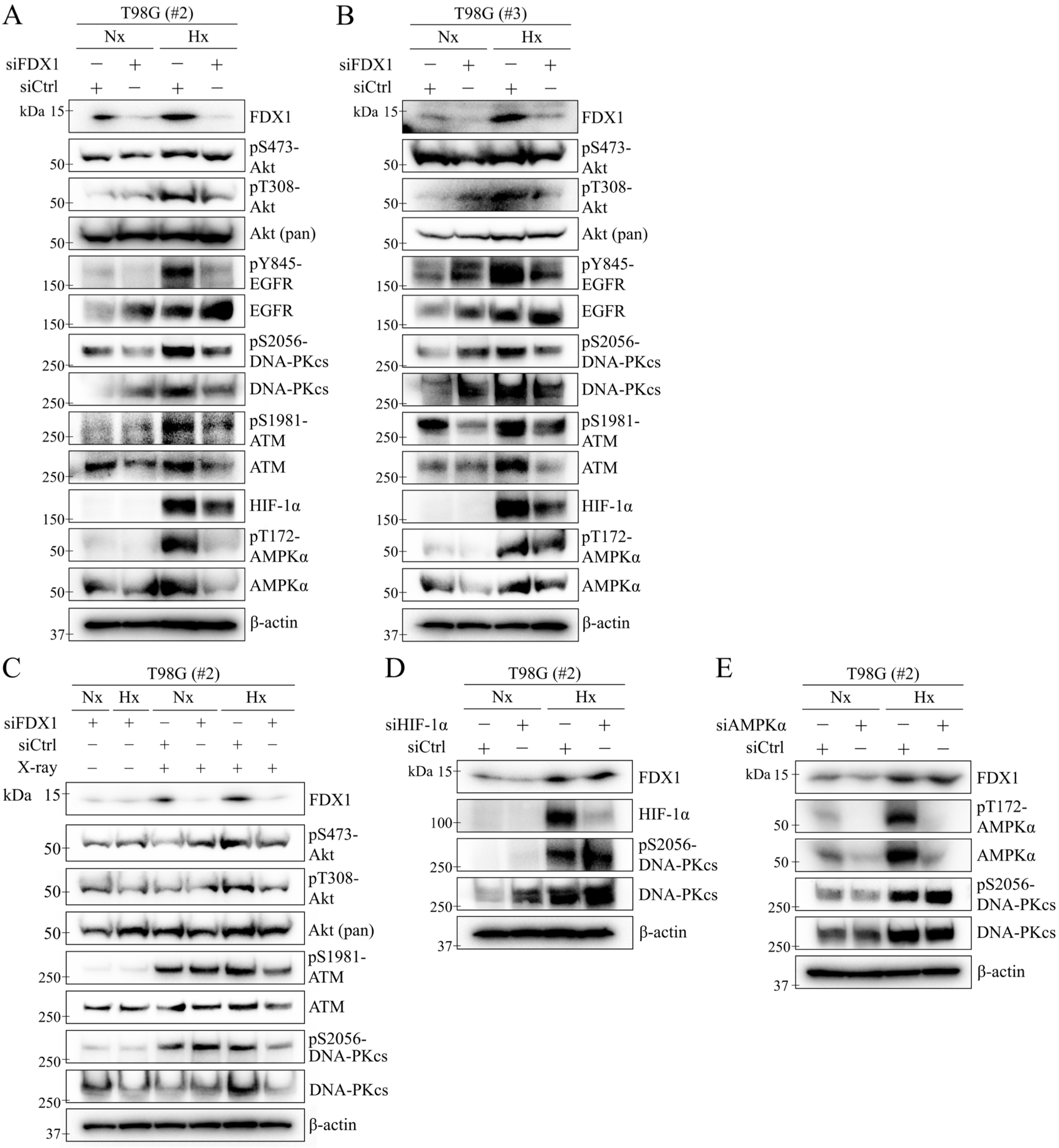

Fig. S1. Verification of Western Blot Reproducibility. Each number ("#") represents an independent biological replicate. (A, B) Replicate experiments corresponding to Fig. 1A, Fig. 3A, and Fig. 5A. (C) Replicate experiments corresponding to Fig. 4. (D) Replicate experiments corresponding to Fig. 5B. (E) Replicate experiments corresponding to Fig. 5C. β-actin served as an internal control. "Nx" represents normoxia, and "Hx" represents severe hypoxia.

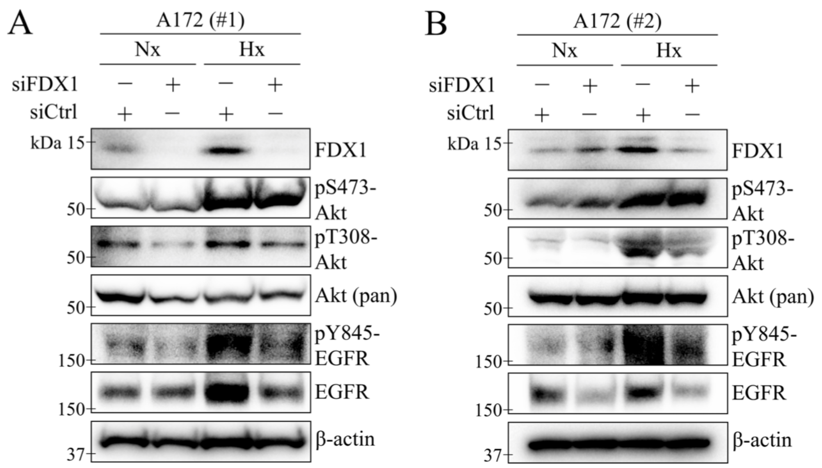

Fig. S2. Induction of FDX1 Expression under Severe Hypoxia and the Effects of FDX1 Knockdown on the Activation of Akt and EGFR in A172. (A) The effects of severe hypoxia on the expression and/or phosphorylation of FDX1, Akt, and EGFR were investigated in A172 cells with FDX1 knockdown. After 48 hours of siRNA treatment (siCtrl or siFDX1), cells were cultured for 18 hours under either severe hypoxia or normoxia and then subjected to Western blot analysis using the indicated antibodies. β-actin served as an internal control. Each number (#) represents an independent biological replicate.

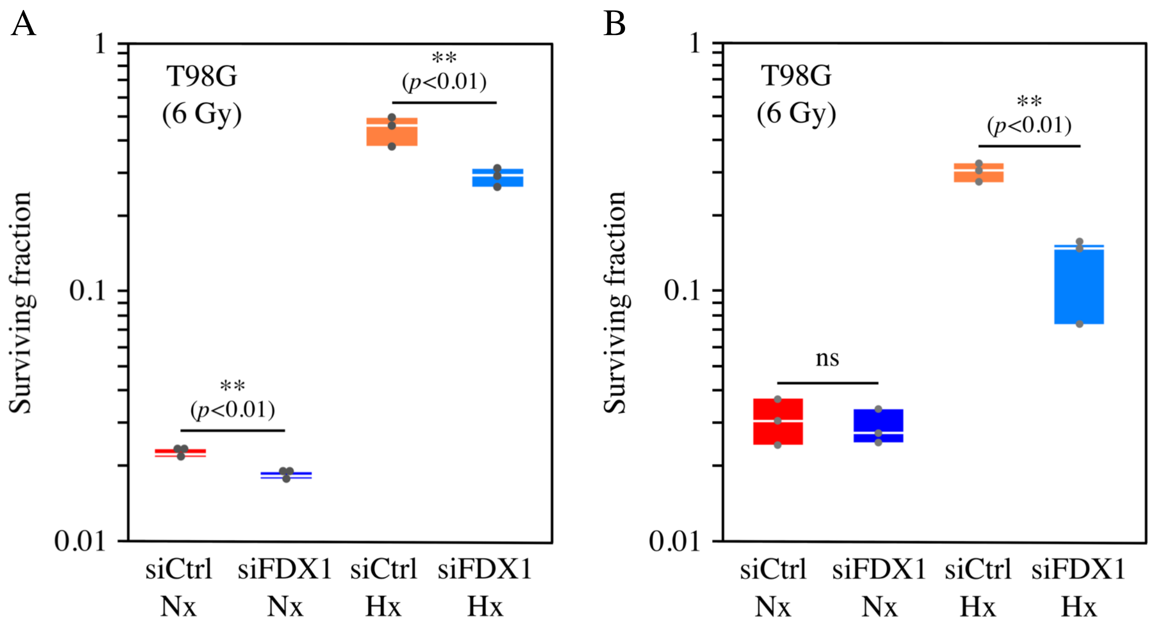

Fig. S3. Verification of Colony Formation Assay. Box plots showing the colony formation assay results (6 Gy) from independent biological replicate experiments corresponding to Fig. 2A. (A) Trial ID #2; (B) Trial ID #3. Values are presented as mean ± SD (n = 3). ns indicates not significant, and $**p < 0.01$ (Student's *t*-test). "Nx" represents normoxia, and "Hx" represents severe hypoxia.

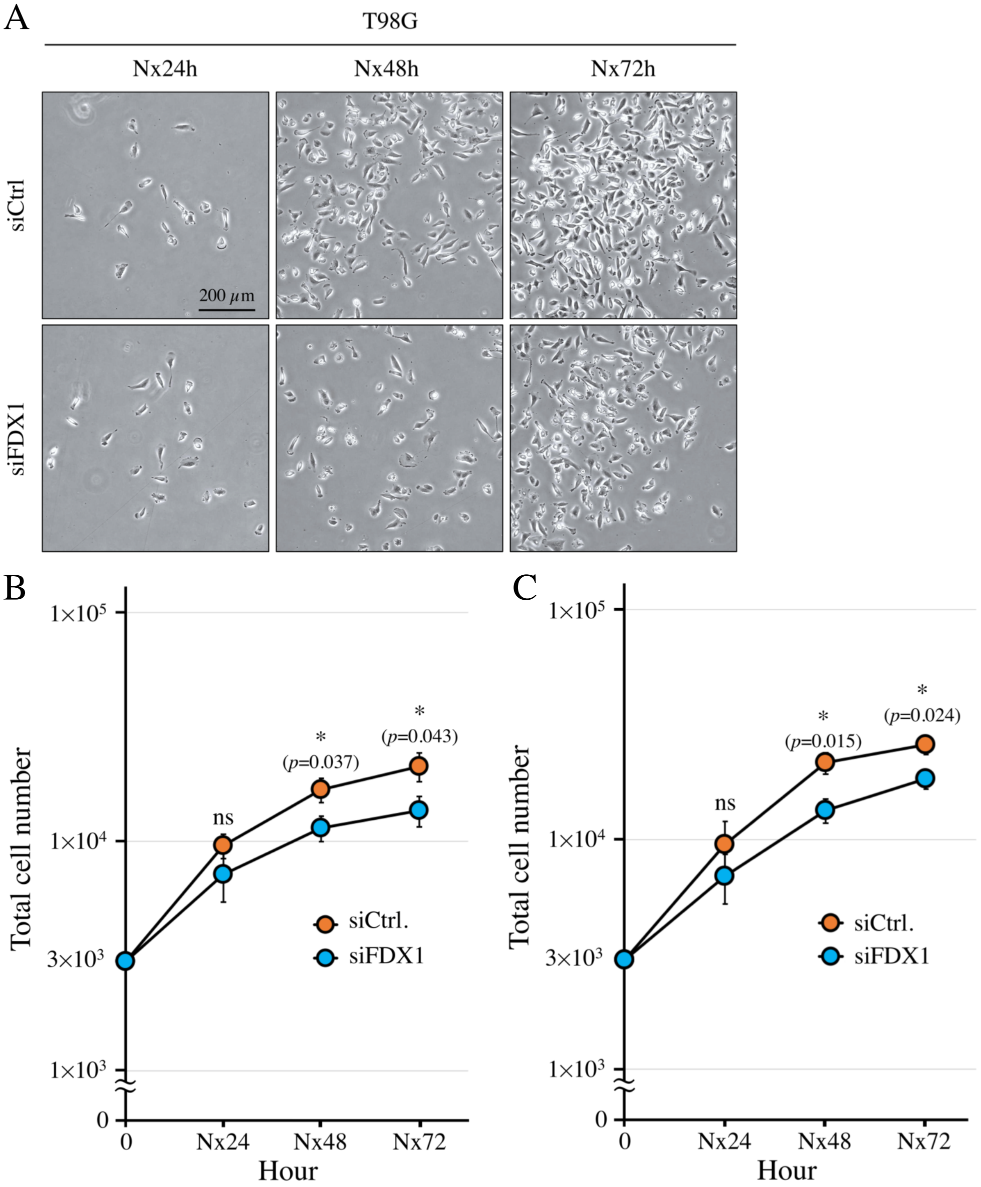

Fig. S4. Effect of FDX1 Knockdown on Cell Proliferation in T98G Cells Under Normoxia. (A) Phase-contrast images and (B) changes in cell number under normoxia (Trial ID #1), and (C) changes in cell number under normoxia (Trial ID #2). Cells were seeded at 3,000 cells per well in a 24-well plate and cultured. Values are presented as mean ± SD (n = 3). *$p < 0.05$ (Student's *t*-test, comparing siCtrl and siFDX1). "ns" indicates not significant. "Nx" represents normoxia.

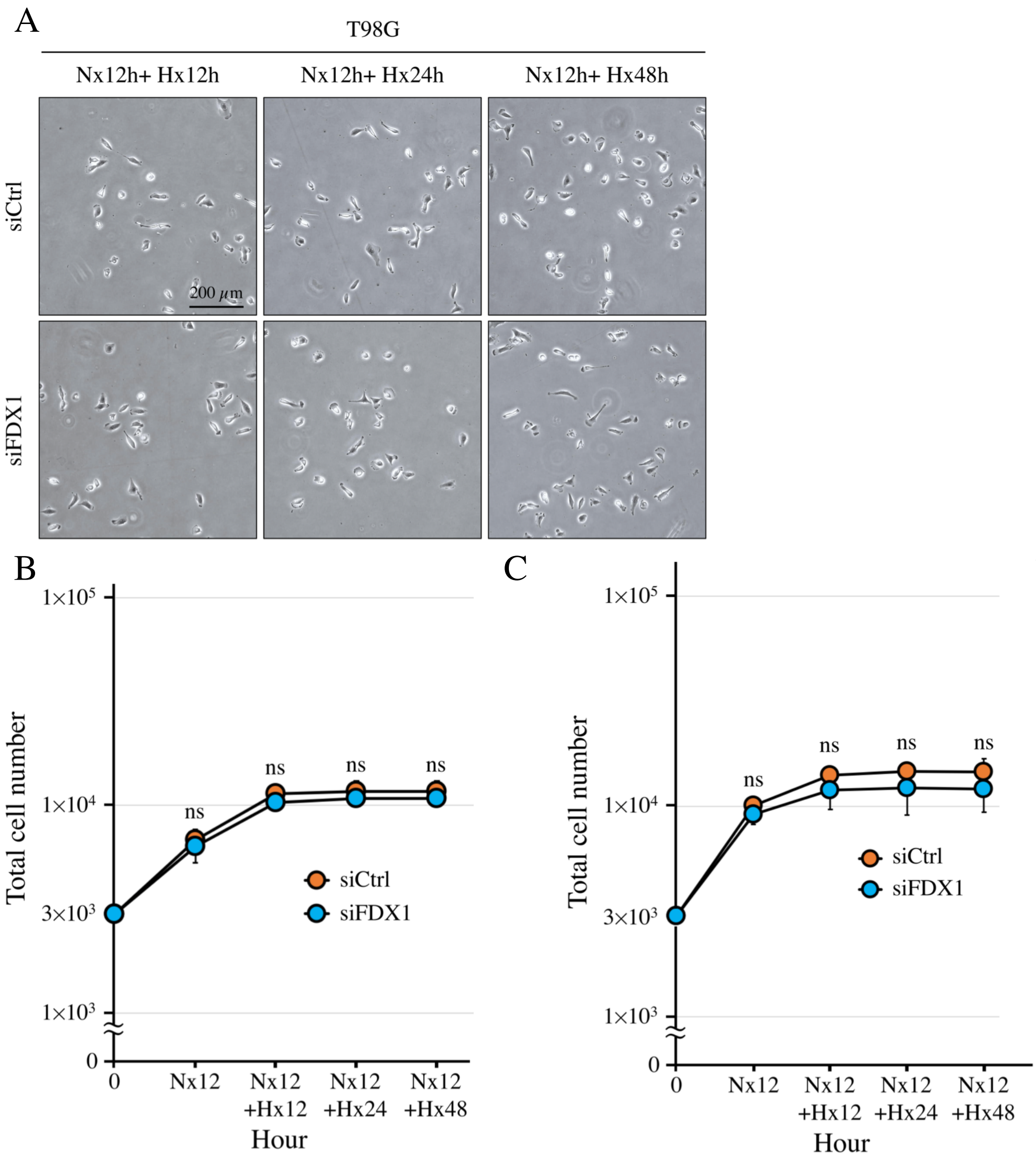

Fig. S5. Effect of FDX1 Knockdown on Cell Proliferation in T98G Cells Under Severe Hypoxia. (A) Phase-contrast images and (B) changes in cell number under normoxia (Trial ID #1), and (C) changes in cell number under normoxia (Trial ID #2). Cells were seeded at 3,000 cells per well in a 24-well plate and cultured. Values are presented as mean ± SD (n = 3), (Student's *t*-test, comparing siCtrl and siFDX1). "ns" indicates not significant. "Nx" represents normoxia, and "Hx" represents severe hypoxia.

Supplementary Table S1. List of antibody for Western blotting

Antibodies	Cat. No.	Vendors
Akt (pan) antibody	4691S	Cell Signaling Technology
Phospho-Akt (S473) antibody	9271S	Cell Signaling Technology
Phospho-Akt (T308) antibody	9275S	Cell Signaling Technology
ATM antibody	NB100-104	NOVUS
Phospho-ATM (S1981) antibody	5883S	Cell Signaling Technology
AMPK antibody	2603S	Cell Signaling Technology
Phospho-AMPK (T172) antibody	2535S	Cell Signaling Technology
DNA-PKcs antibody	SC-9051	Santa Cruz
Phospho-DNA-PKcs (S2056) antibody	ab124918	abcam
Phospho-EGFR (Y845) antibody	2231S	Cell Signaling Technology
EGFR antibody	2232S	Cell Signaling Technology
FDX1 antibody	HPA041630	Sigma
HIF-1 alpha antibody	ab82832	abcam
beta-actin HRP conjugated antibody	ab49900	abcam
Polyclonal Swine Anti-Rabbit immunoglobulins/HRP	P0399	Dako
